# Genetic admixture drives climate adaptation in the bank vole

**DOI:** 10.1038/s42003-024-06549-z

**Published:** 2024-07-15

**Authors:** Michaela Horníková, Hayley C. Lanier, Silvia Marková, Marco A. Escalante, Jeremy B. Searle, Petr Kotlík

**Affiliations:** 1 Laboratory of Molecular Ecology, Institute of Animal Physiology and Genetics, Czech Academy of Sciences, Liběchov, Czech Republic; 2 Department of Biology, Program in Ecology & Evolutionary Biology, University of Oklahoma, Norman, OK USA; 3 Sam Noble Museum, University of Oklahoma, Norman, OK USA; 4 Department of Ecology and Evolutionary Biology, Cornell University, Ithaca, NY USA

**Keywords:** Evolutionary genetics, Evolutionary biology

## Abstract

Genetic admixture introduces new variants at relatively high frequencies, potentially aiding rapid responses to environmental changes. Here, we evaluate its role in adaptive variation related to climatic conditions in bank voles (*Clethrionomys glareolus*) in Britain, using whole-genome data. Our results reveal loci showing excess ancestry from one of the two postglacial colonist populations inconsistent with overall admixture patterns. Notably, loci associated with climate adaptation exhibit disproportionate amounts of excess ancestry, highlighting the impact of admixture between colonist populations on local adaptation. The results suggest strong and localized selection on climate-adaptive loci, as indicated by steep clines and/or shifted cline centres, during population replacement. A subset, including a haemoglobin gene, is associated with oxidative stress responses, underscoring a role of oxidative stress in local adaptation. Our study highlights the important contribution of admixture during secondary contact between populations from distinct climatic refugia enriching adaptive diversity. Understanding these dynamics is crucial for predicting future adaptive capacity to anthropogenic climate change.

## Abstract (web summary)

Genetic mixing among distinct bank vole populations in Britain has enhanced their adaptive diversity for climate adaptation, highlighting the importance of admixture in responding to environmental change.

## Introduction

Standing genetic variation, existing within a species prior to environmental change, plays a critical role in facilitating rapid adaptation by bypassing the time required for new beneficial mutations to arise^1–5^. Admixture between populations of distinct genetic backgrounds accelerates adaptation by introducing pre-existing adaptive variants into the gene pool^6^, yet excessive admixture can disrupt local adaptation^7^. Thus, understanding the consequences of admixture, particularly in the context of climatic variation, provides valuable insights into population responses to ongoing environmental change.

Intraspecific admixture, the result of gene flow between geographically isolated populations of the same species, is commonly associated with postglacial colonization. Surviving the Last Glacial Maximum (LGM; 22–17 kyr) in multiple isolated refugia^8^ likely subjected populations to diverse environmental conditions, promoting adaptive divergence^9^. Upon recolonization, populations from different refugia intermixed, exchanging both neutral and adaptive alleles, generating new allelic combinations with potential for adaptive evolution^2,10^. This standing genetic variation acquired through admixture may have been crucial for postglacial colonization and could also play an important role in future adaptation^11,12^. Understanding the interplay between isolation and admixture sheds light on the mechanisms behind current adaptive variation and improves our ability to anticipate species’ responses to future selection regimes, particularly in the context of global climate change.

Gene flow between divergent populations, particularly involving beneficial adaptive alleles, is predicted to leave genomic imprints in the form of unusual local admixture patterns, where the adaptive alleles of one population may be transferred onto new genomic backgrounds^13^. This may involve one or several alleles conferring an important adaptation^14,15^ or large portions of the genome. Studies show that contact between refugial populations has often resulted in admixture gradients, in which the genome of the population that first colonized a particular area has been partially, or in some cases completely, replaced by genes from a population from another refugium^16–21^. Despite numerous studies investigating secondary contact between populations from different refugia, focusing mainly on neutral variation^22,23^, the exact role of adaptation and selection in this process remains unclear.

Genomic cline analyses, pioneered by Szymura and Barton^24^ and refined by Gompert and Buerkle^25^ within a Bayesian framework (see ref. ^26^ for an alternative implementation), offer a powerful method to assess the interplay between genomic admixture driven by neutral processes and hypotheses involving adaptive gene flow. By examining changes in genotype frequencies along a genome-wide admixture gradient, genomic clines allow for the identification of significant deviations from neutral expectations at individual loci, providing insights into locus-specific admixture patterns^13^. Unlike geographic clines, which track changes in allele frequencies across landscapes, genomic clines predict the probabilities of specific genotypes for a locus as a function of the hybrid index between a pair of species or divergent populations^13^. This index reflects the level of genome-wide admixture between the populations or species under investigation and serves as the neutral expectation against which locus-specific clines are compared^13^. The ability to discern deviations in admixture patterns at individual loci without relying on presupposed geography or admixture history is a key advantage of the genomic clines approach. This versatility enables researchers to unravel complex evolutionary scenarios, from deciphering the dynamics of introgression amidst moving hybrid zones^27–29^ to understanding the role of admixture in facilitating adaptive niche shifts in hybrid taxa^30^.

**Fig. 1:**
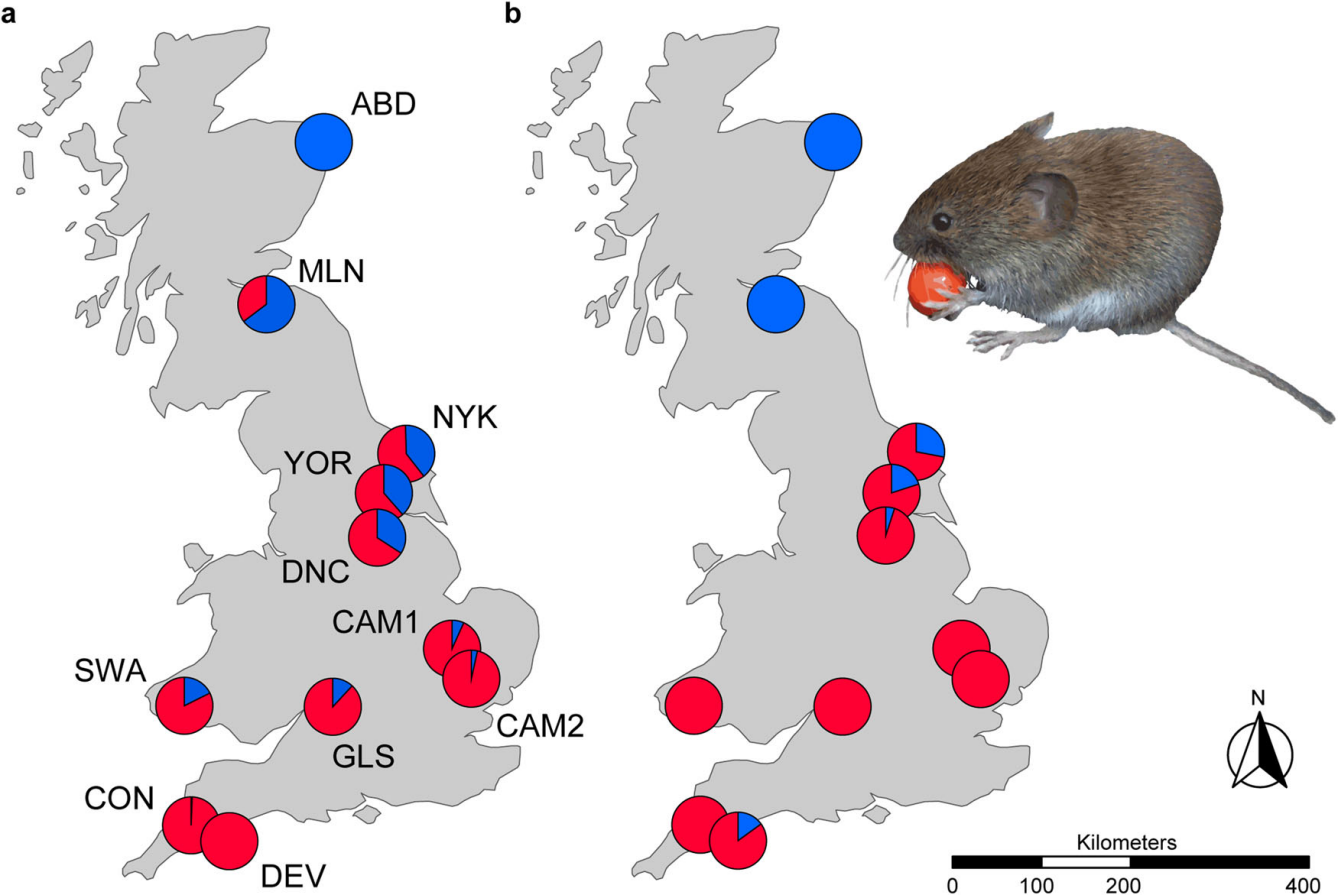
Genomic and genic admixture in British bank voles. **a** Genomic ancestry gradient across 11 localities. Blue and red indicate the respective proportion of ancestry from each colonist as estimated by Admixture analysis. See Supplementary Fig. 2 for details of sampling localities and sample sizes. **b** Distribution of hemoglobin (Hb) allele frequencies, with HbS in blue and HbF in red. The maps were created in R v.4.2.3 using the rnaturalearth package^96^, utilizing the public domain world map data from Natural Earth (https://www.naturalearthdata.com).

The polymorphism at the *HBB-T1* gene, encoding the beta-subunits of the major hemoglobin isoform (hereafter Hb), as found in bank voles (*Clethrionomys glareolus*) in Britain, is a particularly compelling example of the enrichment of adaptive variation resulting from admixture^11,31,32^. Bank voles (and several other vole and shrew species) colonized Britain from Europe at the end of the last glacial period, in a two-stage process from two distinct glacial refugia, resulting in a geographic pattern of genetic variation that remarkably mirrors the Celtic fringe found in humans – the occurrence of Celtic people in the western and northern periphery of Britain^21^. In bank voles this process has been suggested to reflect admixture and partial replacement of the first wave of colonists by a second, following the decline of the first population during the Younger Dryas cold snap, with the original population (the Celtic fringe) restricted to northern and western regions of Britain (particularly Scotland and Wales) while being displaced in the south and east (essentially England)^17,19,21^. British bank voles exhibit a mixture of ancestral genomes derived from two different glacial refugia in mainland Europe, with proportions of unadmixed ancestral genomes increasing toward the north and south of Britain, respectively^19^ (Fig. 1a). In the context of this broad admixture gradient, Hb shows a sharp geographic transition between two functionally distinct allelic variants — HbS and HbF — which are common in the north and south of Britain, respectively^31,32^ (Fig 1b). The two types differ in the presence of a redox-active cysteine in the beta-subunits of HbF, which increases the resistance of erythrocytes to oxidative stress^32,33^, an important source of selection pressure affecting the survival and longevity of organisms under climate change^34–37^. While the Hb is one very well-studied example of intraspecific adaptive variation emerging from admixture of the two colonizing populations of bank voles upon secondary contact in Britain, the implications of this admixture for adaptive variation in other regions of the bank vole genome remain largely unexplored.

The diverse climatic conditions in Britain, spanning a wide range of average annual temperatures (3.1–11.4 °C) and annual rainfall (495–3479 mm), expose bank vole populations to a spectrum of environmental challenges^38^. Prevailing warmer and drier climates in the east and south contrast with cooler and wetter conditions in the west and north^39^, offering an ideal setting to explore the interplay between genetic variation and environmental factors.

To investigate the role of admixture in climate adaptation in British bank voles, we used whole-genome data^40^ from 11 localities across Britain, derived from a previous study^41^. In that earlier study we used the full dataset to determine the capability of local populations to adapt to current and future climatic conditions^41^. Here, we use genomic clines to assess the extent to which local climate adaptation was facilitated by admixture between divergent lineages. Due to computational constraints, we use a smaller dataset than in the earlier study. In this new genomic cline analysis, we identify loci exhibiting admixture between the two colonists of Britain inconsistent with the genome-wide pattern. We contrast these loci with those recovered by a genetic-environmental association analysis to identify candidate adaptive loci for adaptation to different climatic conditions, an approach that has identified loci involved in environmental adaptation across plant and animal species^30,42–46^. If admixture between colonists has introduced variation crucial for local adaptation, we anticipate that loci exhibiting significant excess ancestry from one of the two colonists will be disproportionately represented among candidate loci for climate adaptation^27^. By analyzing the genomic cline for Hb, we test the importance of admixture in the distribution of locally adaptive Hb variability, which has previously been shown to be relevant to population adaptation to global warming^11^. Our results here clearly demonstrate the role of climate-related adaptation during or subsequent to postglacial colonization, including the response at the Hb locus and other genes related to oxidative stress homeostasis. These results suggest that admixture between genomes that diversified in distinct glacial refugia played a key role in facilitating this adaptation process. Importantly, these insights likely hold relevance for future adaptation necessitated by global climate change.

## Results

### Population genomic structure

We used 31,236 genome-wide SNPs for 101 bank voles from 11 localities across Britain^40,41^ (Fig. 1), representing non-admixed populations towards the north (ABD) and south of Britain (DEV), as well as admixed populations along the gradient between these locations, as revealed by previous studies^19,21,41^. Analyses of population structure confirm the existence of the admixture gradient, with the ABD and DEV populations at the extremes (Supplementary Fig. 1). In the ADMIXTURE analysis *K* = 2, *K* = 3 and *K* = 4 showed similar support. At *K* = 2, the northernmost (ABD) and southernmost (DEV) sites were estimated as pure populations showing no admixture, consistent with a two-stage colonization of Britain and partial displacement of bank vole populations originating in different glacial refugia, as suggested by previous studies^19,21^ (Fig. 1), supporting our choice of these populations as representatives of the two colonists in subsequent analyses. At *K* = 3, identified as the optimal *K* for the dataset, the northernmost site (ABD) separated from the northern cluster and at *K* = 4 the southernmost site (DEV) separated from the southern cluster. In both cases the separated sites still showed a high degree of common ancestry with the geographically closest locations in southern Scotland (MLN) and south-western England (CON), respectively, as expected under isolation by distance^19^ (Supplementary Fig. 2).

### SNPs with excess ancestry from one colonist

**Fig. 2:**
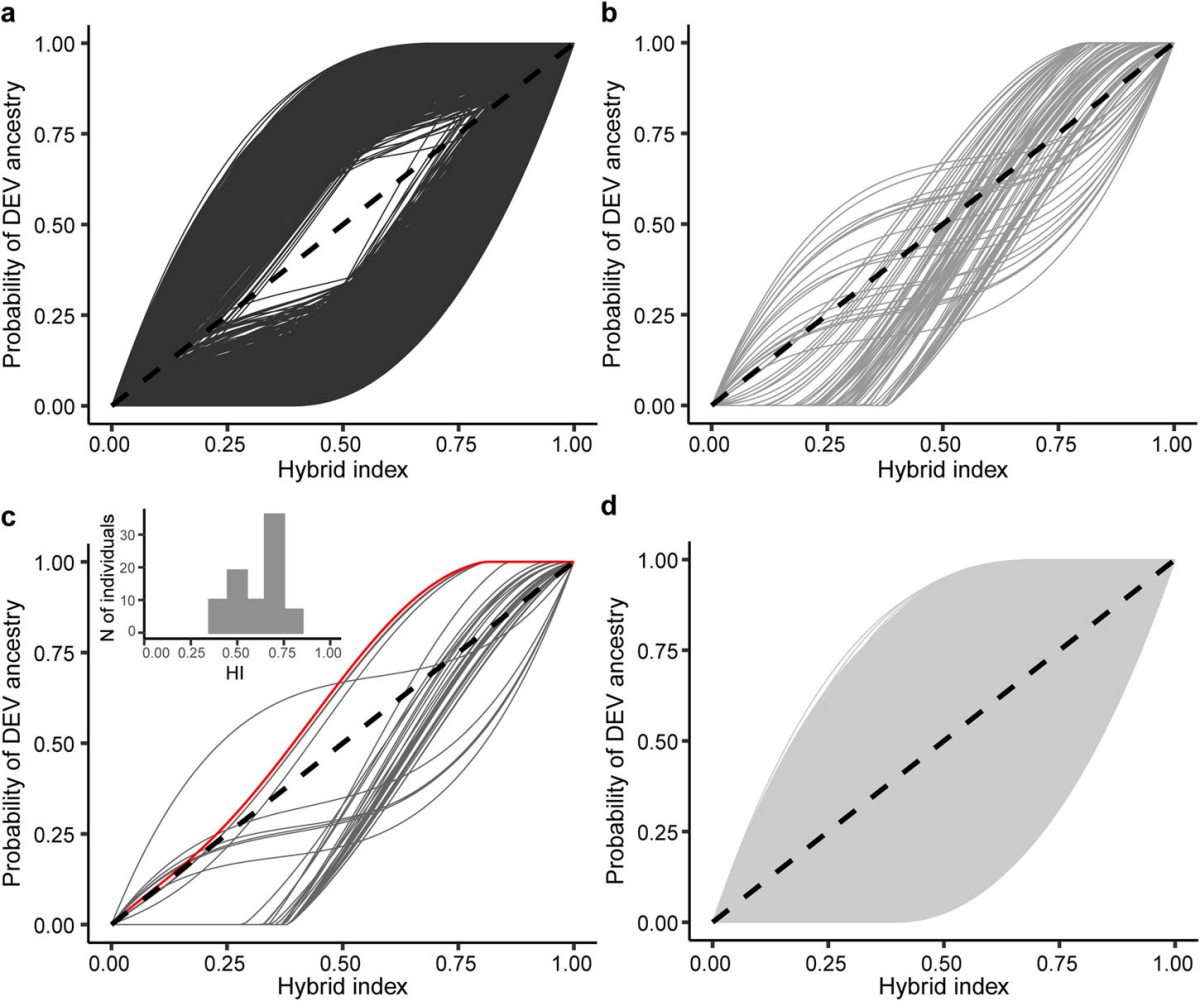
Genomic clines for individual loci. The probability of ancestry from the second colonist (DEV) as a function of the genome-wide hybrid index (HI) for each locus; the dashed line represents the neutral expectation. Each panel shows a different category of loci identified by *bgc*: **a** loci showing excess ancestry for the parameter *α* (cline center), **b** *β* (cline steepness), **c** and both *α* and *β* (with Hb in red), and **d** neutral.

To identify SNP loci exhibiting excess ancestry from one colonizing population relative to the average (admixed) genomic background, we applied *bgc*^47^ to estimate the admixture proportion of an individual’s genome originating from each parental population (i.e., the hybrid index) and identify loci with genomic clines that deviate from this genome-wide pattern based upon posterior probabilities. The *bgc*-estimated values of the hybrid index (i.e., here, the proportion of DEV ancestry) of individuals from the admixed populations (i.e., populations other than ABD or DEV) indicated hybrid indices ranging from 0.38 to 0.81 (Fig. 2c), indicating a genome-wide admixture cline. Patterns of genomic admixture were variable across the genome, with direction of excess ancestry, as indicated by the cline center parameter *α*, being more variable (min = −1.89, max = 1.5) than the rate of change in the cline — the cline steepness parameter, *β* (min = −0.86, max = 1.3).

We identified signatures of locus-specific admixture significantly deviating from the genome wide average admixture (i.e., excess ancestry) in 8132 out of the 31,236 analyzed loci (26%; Fig. 2), 8075 exhibiting excess ancestry from either parental population (i.e., cline center parameter, *α*, significantly different from zero) and only 91 exhibiting increased or decreased rate of admixture (i.e., cline steepness parameter, *β*, significantly different from zero; Table 1). Only 34 loci showed excess ancestry based upon both *α* and *β* parameters (Table 1). The number of loci exhibiting signs of excess DEV ancestry (significantly positive *α*, 4493 loci) was similar to those exhibiting excess ABD ancestry (significantly negative *α*, 3582 loci; Fig. 2a). In contrast, significantly *β*-positive loci (i.e., loci with steeper locus-specific clines) were four times the number of significantly *β*-negative ones (i.e., loci with shallower locus-specific clines; Fig. 2b) as there were 73 and 18 loci, respectively (Table 1). The Hb-locus was identified as a significantly *α*-positive and significantly *β*-positive, indicating higher probability of southern (DEV) ancestry than expected given the genome-wide ancestry, and a significantly steep cline (Fig. 2c). Loci that had similar cline characteristics to Hb (i.e., significantly positive *β* and significant *α*; Fig. 2c) primarily showed an excess of northern (ABD) ancestry (24 loci) and only 4 (including Hb) had an excess of DEV ancestry (Table 1).

**Table 1:** SNP categorization by genomic cline parameters

		All loci		Climate-associated loci	
cline center (*α*)	cline steepness (*β*)	all SNPs	SNPs within or close to genes	all SNPs	SNPs within or close to genes
Negative (ABD)	Negative (shallow)	5 (0.0002)	1 (0.00003)	0 (0)	0 (0)
Negative (ABD)	Not significant	3553 (0.1138)	463 (0.0148)	94 (0.2243)	13 (0.0310)
Negative (ABD)	Positive (steep)	24 (0.0008)	4 (0.0001)	20 (0.0477)	3 (0.0072)
Not significant	Negative (shallow)	12 (0.0004)	2 (0.00006)	0 (0)	0 (0)
Not significant	Not significant	23,104 (0.7397)	2305 (0.0738)	120 (0.2864)	12 (0.0286)
Not significant	Positive (steep)	45 (0.0014)	10 (0.0003)	26 (0.0621)	5 (0.0119)
Positive (DEV)	Negative (shallow)	1 (0.00003)	0 (0)	0 (0)	0 (0)
Positive (DEV)	Not significant	4488 (0.1437)	625 (0.0200)	156 (0.3723)	26 (0.0621)
Positive (DEV)	Positive (steep)	4 (0.0001)	1 (0.00003)	3 (0.0072)	1 (0.0024)

The entire set of 31,236 SNPs, along with a subset of 419 climate-associated SNPs, are grouped based on the estimated cline center (*α*) and steepness (*β*). A cline parameter (*α* or *β*) was considered significant if its 99% credibility interval did not overlap zero. The number of SNPs and the number of SNPs in the coding region of genes or within the 1000 bp flanking region of these genes are shown, with the proportion of SNPs (of the entire dataset or climate-associated loci) in parentheses.

### SNPs associated with environmental conditions

To investigate the interplay between admixture and local adaptation to climate, we used redundancy analysis (RDA) to independently identify candidate adaptive SNP loci associated with climate variation. The RDA approach is an effective method for identifying loci that exhibit unusually strong correlation with environmental variables, suggesting that the alleles provide a selective advantage in a particular environment^48^. The RDA was significant (*p* = 0.001); the first 3 axes explained 100% of the adaptive genetic variance among populations and were retained for the outlier detection, where 419 loci were identified as candidate adaptive loci along the three bioclimatic axes: annual mean temperature, isothermality, and annual precipitation. Of the loci identified as candidates for climate adaptation by the RDA, 299 (71%) also displayed locus-specific admixture deviating from the genome wide average admixture, as indicated by the *bgc* results (i.e., significant *α* and/or *β* parameter; Table 1, Supplementary Fig. 3). This proportion is significantly higher than expected by chance, as only 26% of all loci showed excess ancestry (expected = 26%, observed = 71%, *n* = 419, one-tailed binomial test *p* < 0.001). Among the climate-related loci exhibiting excess ancestry from one colonist (significant *α*), significantly more loci showed excess DEV ancestry than those showing excess ABD ancestry (expected = 50%, observed = 58%, *n* = 273, one-tailed binomial test *p* = 0.004). Notably, out of the 28 loci with significantly steep clines (significantly positive *β*) and shifted centers (significant *α*), 23 were also identified as candidates for climate adaptation by RDA. Among these, 20 exhibited excess ABD ancestry, while 3, including Hb, displayed excess DEV ancestry (Table 1). Post-hoc tests of population differentiation at these loci with shifted and steep clines show that all these loci are F_ST_ outliers.

### Climate-associated genes with excess ancestry

To explore the potential functional importance of candidate climate-adaptive loci exhibiting excess ancestry, we performed Gene Ontology (GO) analyses for the intersection of loci identified by *bgc* and RDA. Among the 299 loci, 48 were located in coding and flanking regions of genes, with four of them associated with non-synonymous substitutions. Of these genes, 35 had identifiable *Mus musculus* orthologs (Supplementary Table 1), two of which (*HBB-T1* and *Rreb1*) harbored non-synonymous SNPs. The GO analysis revealed enrichment of terms related to cell components, biological processes, and molecular functions, with glial cell projection, cell morphogenesis, and protein-containing complex binding being the top enriched terms.

Further exploration focused on processes potentially associated with environmentally-induced oxidative stress responses, which identified two genes (*Fyn* and *Kdm6b*) directly implicated in oxidative stress response, one gene related to antioxidant activity (*HBB-T1*), one gene linked to the response to hypoxia (*Dnmt3a*), and four genes involved in actin filament organization (*Svil*, *Myo5b*, *Flii*, and *Iqgap1*). These genes are primarily involved in cellular responses to hydrogen peroxide, peroxidase activity, hypoxia, and actin polymerization or depolymerization.

Among the 23 RDA outliers that exhibited steep clines and shifted centers akin to Hb, four were located in coding and flanking regions of *M. musculus* orthologs. Notably, three of these genes showed excess ABD ancestry (*Ergic1*, *Flii* and *Aifm2*), while only *HBB-T1* exhibited excess DEV ancestry.

## Discussion

Admixture between divergent lineages is increasingly acknowledged as a key driver of adaptive variation, potentially facilitating rapid responses to climate change^49^. Here, we found that nearly three-quarters of candidate loci for climate adaptation in British bank voles, including Hb and other genes related to oxidative stress homeostasis, exhibit locus-specific admixture that deviates significantly from the genome-wide expectation. These results support the role of selection along climatic gradients in driving admixture between the two colonizing populations. Building upon our previous research, which highlighted the importance of current adaptive gradients in shaping future adaptive capacity^11,41^, our study underscores the key role of admixture in not only shaping but also perpetuating these adaptive gradients essential for supporting resilience in the face of climate change. By shedding light on the mechanisms driving adaptation, our results offer crucial insights into how species can cope with and thrive in a rapidly changing environment.

Consistent with findings from other genomic cline analyses not specifically focused on climate^50,51^, we found that the majority of climate-related loci exhibiting unusual admixture display shifts in cline center (Fig. 2). This pattern aligns with the spread of advantageous alleles between lineages^25^. A small number of loci appear to be affected in terms of admixture rate (the *β* parameter), all with steep clines, suggesting they have undergone a sharp transition onto the alternative genomic background^25^, similar to Hb. It has been proposed that when selection acts on multiple loci with varying impacts, loci undergoing the strongest selection will likely exhibit significant *β* parameters, while those under weaker selection may show excess ancestry based on the *α *parameter^25^. Therefore, the presence of both steep clines and shifted cline centers provides compelling evidence of intense and localized selection acting on these loci during the replacement between the two colonists. Post-hoc analyses confirmed that all of the climate-related loci with shifted and steep clines were F_ST_ outliers, as would be expected if they experienced strong selection in local environments. In contrast, loci with only shifted centers may indicate a more gradual or diffuse pattern of selection, potentially reflecting a lesser degree of environmental pressure.

Most climate-associated loci show excess ancestry from the second colonists, as might be expected if the second colonist carried alleles that provide an advantage under increasingly warmer conditions^11,19,32,33^. However, approximately 40% loci exhibit excess ancestry from the first colonist, suggesting that both colonists possessed loci conferring adaptive benefit related to local climates which migrated to nearby populations across the contact between the two colonizing populations or failed to be displaced during the replacement of the first colonists by the second. In contrast to the widespread admixture at climate-associated loci between the colonists, only a small fraction (3.7%) of loci exhibiting differential patterns of admixture were also identified as potentially conferring an adaptive benefit to climate (i.e., were identified by the RDA). This suggests that while climate adaptation may increase the rate of admixture at certain loci, not all loci that exhibit differential admixture necessarily confer a climate advantage. Taken together, this provides strong evidence indicating that instances of unusual admixture extend beyond loci directly linked to climate adaptation, but that climate-associated loci are more prone to admixture^52–54^. The results imply that selection processes unrelated to climate or genetic drift also contribute to the observed excess ancestry at specific loci. These findings underscore the intricate interplay of different evolutionary forces in shaping the patterns of admixture observed in our study.

The majority of the climate-associated loci with steep clines and shifted cline centers, likely indicative of intense and localized selection during the replacement between the two colonists, exhibit excess ancestry from the first colonist. In contrast, three loci (including Hb) show admixture in the opposite direction. This pattern aligns with theory on moving hybrid zones, suggesting that introgression due to selection during the replacement is typically more likely to originate from the displaced population than in the opposite direction^55,56^. Our findings thus suggest a scenario of gradual replacement of the first colonists by the second, wherein certain climate-related loci exhibit resistance to displacement of locally adaptive alleles derived from the first colonist. This resistance likely reflects the presence of genes under strong climatic selection, with a few genes admixing from the second colonist into the first colonist. Additionally, this replacement process may have been accompanied by a gradual and diffuse reciprocal admixture of advantageous alleles, potentially indicating lower environmental pressure on those genes. This together suggests that admixture between locally adapted bank vole populations has played a crucial role in their adaptation to present-day climate conditions. Importantly, as temperatures continue to rise, increased admixture into the populations descended from the first colonists, located towards the north of the island, may occur^11^. This offers an opportunity for alleles from the second colonist to enhance the resilience of British bank vole populations to future climate change^41^.

Hb emerges as a prominent example of adaptive admixture as it is one of the four genes with SNPs characterized by steep clines. Remarkably, it is the only one among them that shows admixture from the second colonist. The HbF allele, prevalent throughout southern Britain, enhances red blood cell resistance to damage from reactive oxygen species^32^, potentially reducing susceptibility to oxidative stress in warmer climates, both present and future^11^. Our GO analysis supports the role of Hb in adaptation to oxidative stress, with the *HBB-T1* gene directly implicated in antioxidant activity and involved in intracellular oxidative stress regulation (Supplementary Table 1). Previous studies have elucidated the role of a reactive cysteine in HbF in enhancing the antioxidant capacity of bank vole red blood cells^32,33,57^.

The discovery of two additional genes, *Fyn* and *Kdm6b*, containing candidate adaptive climate-related SNPs and exhibiting excess ancestry, underscores the role of oxidative stress as a selective pressure influencing local adaptation through admixture. *Fyn* encodes a tyrosine-protein kinase involved in the Nrf2-dependent antioxidant defence system^58,59^, and *Kdm6b* participates in redox balance regulation by maintaining glutamate metabolism and glutathione levels^60^. Both genes are further implicated in immune response and inflammation regulation^61–64^, interconnected with oxidative stress^65^. Additionally, a gene involved in the cellular response to hypoxia (*Dnmt3a*) encodes a methyltransferase important for the maintenance of mitochondrial function and oxidative capacity^66^, and four genes (*Svil*, *Myo5b*, *Flii* and *Iqgap1*) regulate actin filament and cytoskeleton organization^67–69^, which is crucial during heat exposure and oxidative stress^70,71^.

Interestingly, the majority of these genes associated with oxidative stress response (*Fyn*, *Kdm6b*) and actin filament organization (*Myo5b*, *Svil* and *Iqgap1*) exhibit excess ancestry from the second colonist, aligning with the pattern observed in Hb. This observation suggests that the second colonist may have exhibited adaptation to the warmer climate by enhancing or fine-tuning mechanisms to combat oxidative stress. Conversely, the other three genes characterized by steep and shifted clines—*Ergic1*, *Flii*, and *Aifm2*, respectively crucial for protein transport, homeostasis regulation, and signaling pathways^72–75^—exhibit excess ancestry from the first colonist. These genes may therefore contain variation from the first colonist, adaptive under the increasingly warm climate, potentially offering insights into the relative importance of variation from different glacial refugia.

Our study highlights the crucial role of genes involved in oxidative stress homeostasis. Oxidative stress, exacerbated by escalating environmental temperatures, emerges as an important proximate selection pressure in future climate change scenarios^34–37^. By highlighting the importance of Hb and identifying several other candidate genes, we deepen our understanding of the mechanisms underlying adaptation to environmental stressors. While only a subset of genes is directly associated with the response to oxidative stress, most candidate adaptive genes are involved in the response to and regulation of systemic or cellular environmental stress (Supplementary Table 1). Further studies are needed to elucidate the extent and mechanisms by which variation in loci responding to oxidative stress and other stressors, influenced by past colonization and admixture, fundamentally contributes to climate adaptation.

While it is common to interpret patterns of locus-specific admixture that deviate significantly from null expectations as evidence of selection^25–30^, it is crucial to acknowledge the limitations in this approach. Factors such as genetic drift, limited gene flow, and small population sizes (probably much smaller than typical for these rodents) can influence gene flow patterns^76^, particularly when admixture is recent (tens of generations) and rare^77^. To address this ambiguity and focus specifically on adaptive admixture, we employed more stringent filtering criteria^78,79^, implemented complementary RDA analyses, and selected loci that were demonstrably associated with genes. This approach enabled us to prioritize loci most closely associated with selection and confirmed environmental associations for a number loci with excess ancestry, providing support for adaptive admixture originating from both ancestral populations^30^. We acknowledge that the wavefront dynamics inherent in the movement of hybrid zones can result in certain loci displaying an apparent excess of ancestry from the displaced population. This phenomenon, often referred to as the ghost-of-introgression-past, may not solely indicate selection but may also reflect demographic history^80^. However, here the majority of the loci exhibiting first colonist ancestry and steep clines (indicative of displacement resistance) were recovered also among the climate associated loci, providing support for involvement of selection in shaping clinal patterns, rather than demographic history alone. Thus, despite the inherent limitations, this approach provides an important enhancement of our understanding of local adaptation mechanisms. As population genetics techniques continue to advance to handle larger datasets, integrating denser genomic coverage into genomic cline analyses will deepen our understanding of adaptive admixture, building upon the foundation laid by our study.

The pace of global climate change is forcing many species and populations to rely on pre-existing genetic variation to survive, but not all will benefit equally. Island populations or populations in fragmented habitats are at increased risk as they have limited access to their climatic optimum and less chance of acquiring adaptive variants through gene flow or admixture. Despite numerous challenges, there is growing interest in the use of admixture as a conservation tool for vulnerable populations, including targeted gene flow strategies^81^. Our and other studies^6,11,41^ have offered important insights into the natural process of admixture in adaptation and the relevant genes. However, our understanding of adaptation mechanisms in natural populations remains limited and contributes to the risks associated with assisted adaptation^82^. Further research into adaptation mechanisms, particularly in relation to available genetic variation and population dynamics, is crucial for developing tailored conservation strategies and improving the resilience of species and populations vulnerable to climate change. Understanding adaptation mechanisms demands genome-scale data from a large number of individuals, which is currently unattainable across multiple species. Hence, prioritizing carefully selected model species is a pragmatic necessity. It is imperative that we recognize that such targeted methods are critical to improving our understanding and effectively addressing the hurdles of climate change adaptation in species conservation.

## Materials and methods

### SNP dataset

We derived our SNP genotype data from a previously available^41^ dataset^40^ but used different and more stringent filtering criteria to develop our final dataset. Marková et al.^41^ generated whole-genome sequences for 101 bank voles from 11 localities in Britain using 150 bp paired-end sequencing on the Illumina NovaSeq 6000 platform at the Oklahoma Medical Research Foundation DNA Sequencing Facility, University of Oklahoma, Norman, USA. Sequencing data generated by Marková et al.^41^ are available in the NCBI Sequence Read Archive under BioProject accession number PRJNA1017835. Samples were demultiplexed, converted into fastq formats in bcl2fastq (v2.20.0.422) and raw sequence data were checked for quality by FastQC (v 0.11.9), adapters and low-quality bases were removed with trimmomatic (v. 0.36). Reads for each individual were mapped to the reference genome using *bwa mem* (v. 0.7.10-r789) and resulting files were sorted and indexed with SAMtools (v.1.6.0). After removing duplicate reads identified with picardtools *MarkDuplicates* (v. 2.18.5-6), SNP calling was performed using SAMtools and BCFtools^41^.

To understand the impact of admixture between the two waves of colonists to Britain, we filtered the SNP dataset^40^ generated by Marková et al.^41^ in a manner similar to Marková et al.^41^ but using more stringent criteria to focus on more ancestry informative loci with lower linkage. This meant removing shorter scaffolds with length under 200kbp, retaining a total of 28 scaffolds. SNPs were further filtered using BCFtools, to remove indels, SNPs with more than 50% of missing genotypes, SNPs inside transposable elements, tandem repeats and inside the areas with mappability score lower than 1. Quality filtering was applied to retain biallelic SNPs with *p*-value < 10^−6^, mean depth >5 and minor allele frequency (MAF) >5% to remove potentially uninformative loci that could affect parameter convergence in subsequent *bgc* analyses^83^. A dataset of SNPs in approximate linkage equilibrium was generated using Plink v 1.9^84^ using the flag -indep-pairwise 50 5 0.5 and retaining SNPs with zero missing genotype rate -geno 0 and a minimum separation of 10k bp.

### Bayesian genomic cline analysis

We first verified that the more stringently filtered dataset was capturing the same population structure as previous work by performing principal component analysis (PCA) of the individual SNP genotypes with EIGENSOFT version 7.2.1^85^. Then, to inform the choice of ancestral and admixed populations for subsequent analyses, we estimated the number of *K* ancestral populations and proportions of the genome derived from those populations for each vole using the maximum likelihood method implemented in ADMIXTURE version 1.2^86^. The value of the parameter *K* was estimated using the ADMIXTURE cross-validation procedure, which was run 10 times with random seeds, each time for values from 1 through 10, with 10 replications for each *K*.

To identify loci with alleles exhibiting excess ancestry from one colonizing population relative to the average genomic background, we used the Bayesian genomic cline approach implemented in *bgc* v.1.0.3^47^. In brief, *bgc* uses the hybrid index, i.e., the admixture proportion of the individual’s genome from one parental population (here, DEV), and the locus-specific genomic cline parameters *α* and *β* to estimate the posterior probability of locus-specific ancestry within the admixed population^25^. The *α* parameter represents the genomic cline center and indicates the direction of excess ancestry. Positive outliers indicate an excess of DEV (second colonist) ancestry, whereas negative outliers indicate an excess of ABD (first colonist) ancestry. The *β* parameter is the genomic cline rate parameter, reflecting the rate of change, where negative values are translated into a shallower genomic cline with higher admixture rate and positive values indicate a steeper cline with a sharper transition between the two parental populations. When both parameters are equal to 0, the locus-specific ancestry and the hybrid index are equivalent and match the neutral genomic background expectation.

Two independent *bgc* runs were performed with 50,000 MCMC steps and every 10th iteration retained, i.e., with 5000 samples obtained from each run. Default settings were used for all other parameters. After run completion, parameter traces were plotted using ClineHelpR^87^ and visually inspected for convergence, the first 1000 samples were discarded as burn-in. Upon checking convergence of the independent runs together, the runs were subsequently combined. Loci with locus-specific admixture different from genome-wide average admixture were identified as those for which the 99% equal-tail probability interval for the posterior probability distribution of the cline parameters *α* or *β* did not overlap zero.

To ascertain whether loci that exhibit shifted and steep genomic clines also exhibit increased population differentiation, we performed a post-hoc F_ST_ test to determine whether these loci have F_ST_ values that exceed the 95th percentile threshold.

### Genetic-environmental association analysis

To assess how the admixture between ancestral populations influences local environmental adaptation (and how local adaptation influences gene flow and admixture) we independently identified loci that may confer an adaptive advantage using RDA. The RDA genetic-environmental association approach aims to identify loci which show an unusually strong correlation with environmental variables, suggesting that the alleles provide a selection advantage in a particular environment^48^. RDA is a two-step analysis in which genetic and environmental data are analyzed using multivariate linear regression, producing a matrix of fitted values. Then, a PCA of the fitted values is used to produce canonical axes, which are linear combinations of the environmental predictors^88^. RDA has been shown to have a low rate of false positives and a high rate of true positives across a range of demographic histories and sampling designs^48^.

To perform RDA in the bank vole we obtained bioclimatic variables from WorldClim v.2^89^ and used the pairs.panels function of the vegan package in R^90^ to select a set of uncorrelated variables based on thresholds *r* < 0.7 and variance inflation factor <10^48,91^. The retained bioclimatic variables were annual mean temperature (BIO 01), isothermality (BIO 03) and annual precipitation (BIO 12). Subsequently, an RDA was run with the three bioclimatic variables as predictors in the vegan package. To control for false discovery rate (FDR), we used the qvalue R package to convert the *p*-values to *q*-values. The candidate adaptive loci were selected with an FDR threshold of 0.01 to minimize false positives.

The number of adaptive loci in different categories, as well as their comparison to random expectations, was assessed using a one-sided binomial test.

### Identification of candidate adaptive genes

To investigate the possible functional significance of the candidate adaptive loci exhibiting signs of adaptive admixture, we performed GO analyses for the intersection of the loci identified by *bgc* and RDA. The QIAGEN CLC Genomic workbench software was used to identify SNPs in the bank vole genes and 1000 bp long flanking region on either side. Then for each *C. glareolus* gene, we identified the orthologous *Mus musculus* gene using the UniProt database^92^. Panther v.17.0^93^ was used to analyze enrichment for GO biological processes, molecular functions, pathways and reactome pathways^94^, identifying functional categories enriched at a false discovery rate-adjusted *p*-value threshold of 0.05. To see if there are other loci that could have similar functional importance related to mediation of oxidative stress, as was demonstrated for Hb in bank voles, we followed the approach of Marková et al.^41^. Thus, we used QuickGO v 2022-11-18^95^ to detect involvement of the genes in particular sub-processes related to organismal response to environmentally (particularly temperature) induced oxidative stress, as defined by specific GO terms (response to oxidative stress (GO:0006979), response to temperature stimulus (GO:0009266), response to hypoxia (GO:0001666) and actin filament organization (GO:0007015)).

### Statistics and reproducibility

Our study made use of the SNP dataset for 101 bank voles from 11 localities in Britain (Supplementary Fig. 1), generated by Marková et al.^41^, and accessible through the Dryad Digital Repository^40^. To understand the impact of admixture between the two waves of colonists to Britain, we filtered the data using more stringent criteria to focus on more ancestry informative loci with lower linkage, as detailed within the Methods section SNP dataset. All subsequent analyses were performed using a publicly available software and packages available in R. Bioclimatic variables for the RDA analysis were obtained from publicly available data in the WorldClim database^89^. The rationale for the analyses, as well as the software, packages and parameter settings used for the analyses are detailed within their respective Methods sections. The maps were created in R v.4.2.3 using the rnaturalearth package^96^, utilizing the public domain world map data from Natural Earth (https://www.naturalearthdata.com). The figures were assembled and labeled in Adobe Illustrator, utilizing the original graphical output from the respective analysis packages.

### Reporting summary

Further information on research design is available in the Nature Portfolio Reporting Summary linked to this article.

### Supplementary information

Supplementary information

Description of Additional Supplementary Files

Supplementary Data 1

Reporting summary

## Data Availability

The SNP dataset of Marková et al.^41^, from which the dataset used in this study was derived, as described in the corresponding Methods section, is accessible through the Dryad Digital Repository (10.5061/dryad.kwh70rz96)^40^. The source data for Fig. 2 are in Supplementary Data 1.
