# Supplementary material for: Genetic admixture drives climate adaptation in the bank vole
Source: Commun Biol. 2024 Jul 15;7:863. doi: 10.1038/s42003-024-06549-z (PMC11251159; doi:10.1038/s42003-024-06549-z)
Supplement: Supplementary file 1 — Supplementary information [file 42003_2024_6549_MOESM1_ESM.pdf]

Supplementary information

# Genetic admixture drives climate adaptation in the bank vole

Michaela Horníková[1], Hayley C. Lanier[2,3], Silvia Marková[1], Marco Escalante[1], Jeremy B. Searle[4] & Petr Kotlík[1]

[1] Laboratory of Molecular Ecology, Institute of Animal Physiology and Genetics, Czech Academy of Sciences, Liběchov, Czech Republic

[2] Department of Biology, Program in Ecology & Evolutionary Biology, University of Oklahoma, Norman, OK, USA

[3] Sam Noble Museum, University of Oklahoma, Norman, OK, USA

[4] Department of Ecology and Evolutionary Biology, Cornell University, Ithaca, NY, USA

Correspondence: Petr Kotlík, kotlik@iapg.cas.cz

## Supplementary Figures

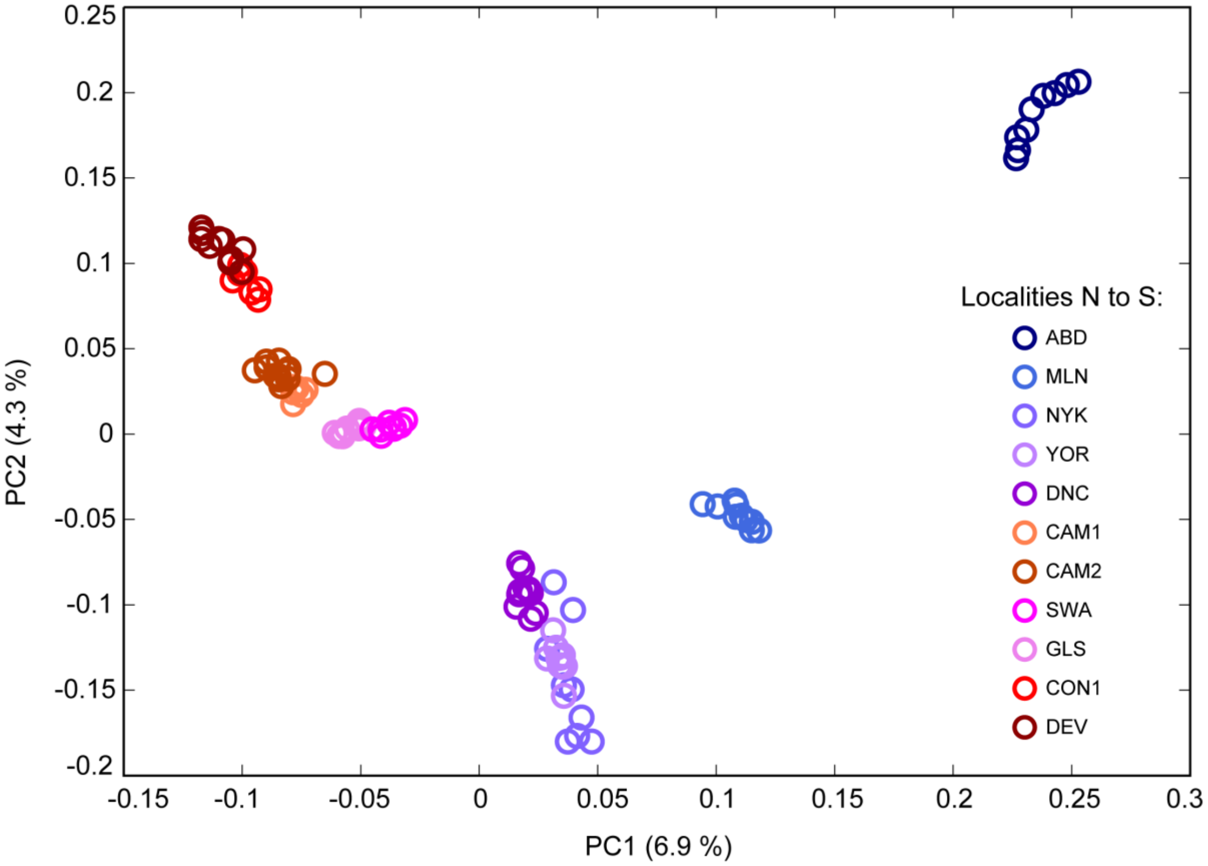


**Supplementary Fig. 1. PCA distribution of individuals from 11 British localities across the admixture gradient.** Non-admixed populations ABD (dark blue) and DEV (dark brown-red) are positioned at the extremes of the gradient, with admixed populations scattered between them along PC1.

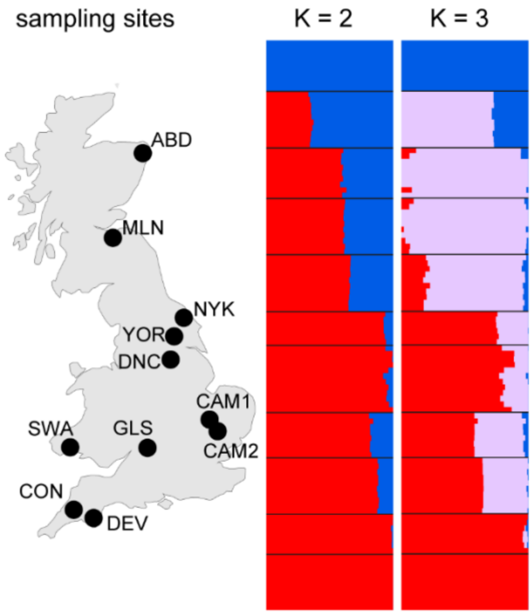


| code | locality | lat. | long. | N | southern ancestry | HbF freq. |
|---|---|---|---|---|---|---|
| ABD | Maud, Aberdeenshire | 57.48 | -2.08 | 9 | 0.00 | 0.00 |
| MLN | Roslin, Midlothian | 55.84 | -3.10 | 10 | 0.35 | 0.00 |
| NYK | Pickering, North Yorkshire | 54.29 | -0.68 | 9 | 0.60 | 0.72 |
| YOR | Heslington, North Yorkshire | 53.93 | -1.01 | 10 | 0.61 | 0.80 |
| DNC | Doncaster, South Yorkshire | 53.48 | -1.13 | 10 | 0.66 | 0.95 |
| CAM1 | Wicken, Cambridgeshire | 52.28 | 0.27 | 6 | 0.93 | 1.00 |
| CAM2 | Temple End, Cambridgeshire | 52.13 | 0.41 | 12 | 0.97 | 1.00 |
| SWA | Llanddowror, Carmarthenshire | 51.78 | -4.56 | 8 | 0.82 | 1.00 |
| GLS | Cirencester, Gloucestershire | 51.77 | -1.92 | 10 | 0.88 | 1.00 |
| CON | Trebartha, Cornwall | 50.57 | -4.44 | 7 | 0.99 | 1.00 |
| DEV | Diptford, Devon | 50.41 | -3.76 | 10 | 1.00 | 0.85 |

**Supplementary Fig. 2. Sample localities and ADMIXTURE plots.** Bar plots represent admixture proportions for K=2 (indicating 2-phase colonization) and K=3 (best supported, similar to Marková et al.[1]). Each horizontal bar represents an individual, with colours indicating admixture proportions based on the respective K value. The table provides information on sample localities and numbers, admixture proportion of southern (DEV) ancestry, and HbF allele frequency. The map was created in R v.4.2.3, using the rnaturalearth package2 containing publicly available world map data from Natural Earth.

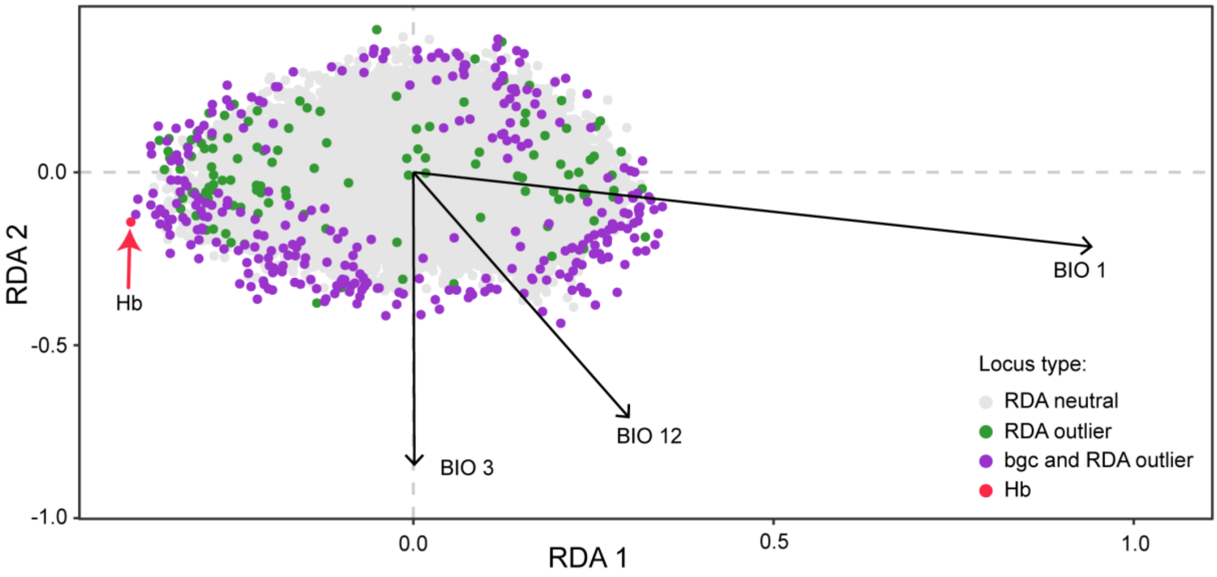


**Supplementary Fig. 3. Genetic-environmental association analysis.** The Redundancy Analysis (RDA) plot illustrates the projection of loci and environmental variables onto the first two axes. Candidate climate-adaptive loci are highlighted in green and purple, with purple indicating climate-adaptive loci exhibiting excess ancestry as identified by Bayesian genomic cline analysis. The haemoglobin locus, emphasised in red, is one of the candidate adaptive loci with excess ancestry.

**Supplementary Figures references:**


1. Marková, S. *et al.* Local adaptation and future climate vulnerability in a wild rodent. *Nat. Commun.* **14**, 7840 (2023).
2. Massicotte, P. & South, A. rnaturalearth: World Map Data from Natural Earth. (2024).

## Supplementary Table

**Supplementary Table 1. Overview of candidate adaptive genes functions potentially linked to environmentally induced systemic or cellular stress regulation.** It includes climate-associated loci exhibiting excess ancestry from either colonist, located within or within 1000 bp of coding regions of genes, along with corresponding *Mus musculus* ortholog information. For each locus, possible function of the gene or its product in regulation of environmentally induced stress is provided. The list aims to enhance our understanding of the adaptively admixed climate-related genes in British bank voles. However, it may not be exhaustive, as many genes exhibit multifunctionality. Genes identified as involved in processes related to oxidative stress response, as defined by particular Gene Ontology (GO) terms, are marked with an asterisk (*).

| SNP | Cline centre α | Cline slope β | UniProt accession | Gene | Protein name | Functions potentially linked to environmentally induced systemic and cellular stress regulation |
|---|---|---|---|---|---|---|
| chr1:101229696 | pos | NA | Q8VHA6 | *Asb18* | Ankyrin repeat and SOCS box protein 18 | ASB18 function not fully characterised; ASB proteins mediate ubiquitination[1], important for cellular stress response[2,3] |
| chr1:50317140 | pos | NA | P39688 | *Fyn** | Tyrosine-protein kinase Fyn | Nrf2 dependent oxidative stress response regulation[4,5]; stress erythropoiesis regulation[6]; immune response regulation via T cell activation, development and signalling regulation[7] and inflammatory response mediation[8,9]; cell survival promotion via apoptosis prevention[10] |
| chr2:114684072 | neg | NA | O88866 | *Hunk* (syn. *Makv*) | Hormonally up-regulated neu tumor-associated kinase | intracellular homeostasis maintenance via autophagy regulation / support[11,12] |
| chr3:100390653 | pos | NA | Q3UV71 | *Tmtc1* | Protein O-mannosyl-transferase TMTC1 | endoplasmatic reticulum calcium homeostasis and signalling regulation[13], thus possibly playing role in Ca2+ dependent cell death / apoptosis regulation[14] |
| chr3:69668711 | pos | NA | Q9R0K7 | *Atp2b2* (syn. *Pmca2*) | Plasma membrane calcium-transporting ATPase 2 | cellular $Ca^{2+}$ homeostasis[15]; $Ca^{2+}$ dependent cell death / apoptosis regulation[16] |
| chr4:581079 | pos | NA | P70207 | *Plxna2* (syn. *Kiaa0463*) | Plexin-A2 | neuroinflammation regulation[17]; cell survival and proliferation[18] |
| chr4:66447603 | pos | NA | P15105 | *Glul* | Glutamine synthetase | glutamine synthesis, glutamate metabolism[19]; (glutamine plays role in redox balance maintenance by fuelling glutathione synthesis[20] and cell survival under physiological stress by autophagy induction[21])); inflammation response regulation[22]; ammonia homeostasis maintanance[23] |
| chr4:85547634 | pos | NA | Q810U3 | *Nfasc* | Neurofascin | stress coping regulation[24]; neuronal development and signalling[25,26] |
| chr6:102632294 | NA | pos | O88508 | *Dnmt3a** | DNA (cytosine-5)-methyltransferase 3A | intracellular oxidative stress regulation[27,28]; inflammatory response regulation[29,30] |
| chr7:38734860 | pos | NA | Q60980 | *Klf3* (syn. *Bklf*) | Krueppel-like factor 3 | adipogenesis regulation[31]; inflammation regulation[32,33] |
| chr8:2995545 | pos | pos | P02088 | *Hbb-t1** | Hemoglobin subunit beta-1 | intracellular oxidative stress regulation[34–36] |
| chr8:48110813 | pos | NA | Q920L1 | *Fads1* | Acyl-CoA (8-3)-desaturase | polyunsaturated fatty acid metabolism regulation[37] |
| chr8:97801733 | pos | NA | P50283 | *Cd7* | T-cell antigen CD7 | immune response modulation via t-cell apoptosis regulation[38]; regulatory T-cell homeostasis maintanance[39] |

| SNP | Cline centre α | Cline slope β | UniProt accession | Gene | Protein name | Functions potentially linked to environmentally induced systemic and cellular stress regulation |
|---|---|---|---|---|---|---|
| chr9:14542442 | neg | pos | Q9DC16 | *Ergic1* (syn. *Ergic32*) | Endoplasmic reticulum-Golgi intermediate compartment protein 1 | Ergic1 function not well characterised, possibly innate immunity regulation[40]; endoplasmatic reticulum proteostasis maintenance via membrane traffic and cargo transport regulation[41], possibly regulating redox homeostasis[42] |
| chr9:41157932 | neg | pos | Q9JJ28 | *Flii** (syn. *Fli1, Fliih*) | Protein flightless-1 homolog | actin polymerization[43] and actin filament organization regulation[44]; $Ca^{2+}$ homeostasis, $Ca^{2+}$ dependent cell death / apoptosis regulation[45]; inflammatory response regulation[46,47] |
| chr9:41928124 | pos | NA | Q9R1S0 | *B9d1* (syn. *Eppb9*) | B9 domain-containing protein 1 | regulation of ciliogenesis[48,49] involved in cellular stress and DNA damage response[50–52] |
| chr9:49602944 | pos | NA | Q5NCY0 | *Kdm6b** (syn. *Jmjd3, Kiaa0346*) | Lysine-specific demethylase 6B | redox balance regulation - glycolysis, glutamate metabolism, and glutathione levels maintanance[53]; HIF-1 mediated hypoxia response regulation[54]; immune response, inflammation and cellular senescence regulation[55] |
| chr9:77534403 | neg | NA | Q91ZV7 | *Plxdc1* (syn. *Tem7*) | Plexin domain-containing protein 1 | Pigment Epithelium Derived Factor neuroprotective effect mediation[56] |
| chr9:80592326 | pos | NA | O35047 | *Psmc3ip* (syn. *Hop2, Tbpip*) | Homologous-pairing protein 2 homolog | apoptosis regulation[57]; transcription regulation[58,59]; DNA repair through homologous recombination regulation[60] |
| chr9:83743313 | neg | NA | P10637 | *Mapt* (syn. *Mtapt, Tau*) | Microtubule-associated protein tau | cellular DNA protection from stress induced damage[61,62]; gene expression regulation[63] |
| chr10:22658859 | neg | NA | Q8BLX4 | *Slc35c1* (syn. *Fuct1*) | GDP-fucose transporter 1 | immune response modulation via regulatory T cell function regulation[64]; cell proliferation and inflammatory response modulation[65,66] |
| chr12:2521151 | pos | NA | Q8K4L3 | *Svil** | Supervillin | actin cytoskeleton and filament organization[67,68]; cell survival regulation[69]; immune response regulation via natural killer cells function regulation[70]; participation in inflammatory response[71] |
| chr12:52187560 | pos | NA | Q3UH06 | *Rreb1* | Ras-responsive element-binding protein 1 | DNA damage response and apoptosis regulation[72], transcription regulation[73,74]; cell proliferation regulation[75] |
| chr13:7182807 | pos | NA | P21271 | *Myo5b** (syn. *Kiaa1119*) | Unconventional myosin-Vb | actin-based processive motor[76] - membrane traffic regulation, exocytosis[77,78] |
| chr13:7793294 | neg | NA | O35253 | *Smad7* (syn. *Madh7, Madh8*) | Mothers against decapentaplegic homolog 7 | apoptosis regulation[79,80]; inflammation modulation[81,82] |
| chr15:18429814 | pos | NA | Q9CT81 | *Elp1* (syn. *Ikap, Ikbkap*) | Elongator complex protein 1 | elongator plays role in transcriptional elongation[83,84]; tRNA modification[85,86] |
| chr16:38078910 | NA | pos | Q9JHU2 | *Palmd* | Palmdelphin | DNA damage induced apoptosis regulation[87]; muscle regeneration[88]; glycolysis and NF-κB–mediated inflammation response regulation[89] |

| SNP | Cline centre α | Cline slope β | UniProt accession | Gene | Protein name | Functions potentially linked to environmentally induced systemic and cellular stress regulation |
|---|---|---|---|---|---|---|
| chr16:50843108 | neg | NA | Q922J6 | *Tspan2* | Tetraspanin-2 | immune response and CNS autoimmunity regulation[90]; neuroinflammation suppresion[91] |
| chr18:40014222 | neg | pos | Q8BUE4 | *Aifm2* (syn. *Amid*) | Apoptosis-inducing factor 2 | mitochondrial stress signalling and apoptosis regulation[92,93]; cold and diet induced thermogenesis regulation[94] |
| chr18:47069096 | neg | NA | G5E8K5 | *Ank3* | Ankyrin-3 | stress coping regulation[95], possibly impacting stress-modulated longevity[96]; neuronal development and signalling[97,98] |
| chr21:13873764 | pos | NA | Q9WTR5 | *Cdh13* | Cadherin-13 | endothelial cell protection from stress-induced apoptosis[99,100]; adiponectin-mediated protective effect regulation in cardiovascular system[101] and skeletal muscle[102]; blood pressure and stamina regulation[103] |
| chr21:33817108 | neg | NA | Q80TS5 | *Znf423* (syn. *Ebfaz*, *Kiaa0760*, *Nur12*, *Oaz Zfp423*) | Zinc finger protein 423 | hypoxia induced autophagy regulation[104]; DNA damage response regulation[105,106]; adipocyte development[107] and thermogenesis regulation[108] |
| chr23:15039066 | pos | NA | Q9JKF1 | *Iqgap1** | Ras GTPase-activating-like protein IQGAP1 | actin assembly and actin cytoskeleton organization regulation, actin filament cross-linking[109–111] ; stress response mediation of alternative splicing regulators[112]; NF-κB–mediated inflammation response regulation[113] |
| chr25:4709053 | neg | NA | P15327 | *Bpgm* | Bisphosphoglycerate mutase | tissue hypoxia and inflammation prevention via 2,3-BPG production induced $0^2$ release capacity increase[114]; serine homeostasis regulation[115], serine is important for redox balance, immune response or lipid metabolism[116] |
| chr27:26278004 | pos | NA | B2RY04 | *Dock5* (syn. *Lr2*, *Rlc*) | Dedicator of cytokinesis protein 5 | immune response modulation via neutrophile chemotaxis, ROS production and extracellular trap formation regulation[117]; energy balance and glucose homeostasis regulation[118]; lipid metabolism regulation[119] |

**Supplementary Table 1 references:**


1. Linossi, E. M. & Nicholson, S. E. The SOCS box—Adapting proteins for ubiquitination and proteasomal degradation. *IUBMB Life* **64**, 316–323 (2012).
2. Maxwell, B. A. *et al.* Ubiquitination is essential for recovery of cellular activities after heat shock. *Science* **372**, eabc3593 (2021).
3. Sheng, X., Xia, Z., Yang, H. & Hu, R. The ubiquitin codes in cellular stress responses. *Protein Cell* pwad045 (2023) doi:10.1093/procel/pwad045.
4. Lee, N. *et al.* The role of fucoxanthin as a potent Nrf2 activator via Akt/GSK-3β/Fyn axis against amyloid-β peptide-induced oxidative damage. *Antioxidants* **12**, 629 (2023).
5. Mathur, A., Rizvi, F. & Kakkar, P. PHLPP2 down regulation influences nuclear Nrf2 stability via Akt-1/Gsk3β/Fyn kinase axis in acetaminophen induced oxidative renal toxicity: Protection accorded by morin. *Food Chem. Toxicol.* **89**, 19–31 (2016).
6. Beneduce, E. *et al.* Fyn kinase is a novel modulator of erythropoietin signaling and stress erythropoiesis. *Am. J. Hematol.* **94**, 10–20 (2019).

7. Salmond, R. J., Filby, A., Qureshi, I., Caserta, S. & Zamoyska, R. T-cell receptor proximal signaling via the Src-family kinases, Lck and Fyn, influences T-cell activation, differentiation, and tolerance. *Immunol. Rev.* **228**, 9–22 (2009).
8. Saminathan, H. *et al.* Fyn kinase mediates pro-inflammatory response in a mouse model of endotoxemia: relevance to translational research. *Eur. J. Pharmacol.* **881**, 173259 (2020).
9. Mkaddem, S. B. *et al.* Lyn and Fyn function as molecular switches that control immunoreceptors to direct homeostasis or inflammation. *Nat. Commun.* **8**, 246 (2017).
10. Tang, X., Feng, Y. & Ye, K. Src-family tyrosine kinase fyn phosphorylates phosphatidylinositol 3-kinase enhancer-activating Akt, preventing its apoptotic cleavage and promoting cell survival. *Cell Death Differ.* **14**, 368–377 (2007).
11. Zambrano, J. N. *et al.* HUNK phosphorylates rubicon to support autophagy. *Int. J. Mol. Sci.* **20**, 5813 (2019).
12. Zhou, C. *et al.* Integral membrane protein 2A inhibits cell growth in human breast cancer via enhancing autophagy induction. *Cell Commun. Signal.* **17**, 105 (2019).
13. Sunryd, J. C. *et al.* TMTC1 and TMTC2 are novel endoplasmic reticulum tetratricopeptide repeat-containing adapter proteins involved in calcium homeostasis. *J. Biol. Chem.* **289**, 16085–16099 (2014).
14. Marchi, S. *et al.* Mitochondrial and endoplasmic reticulum calcium homeostasis and cell death. *Cell Calcium* **69**, 62–72 (2018).
15. Brini, M., Calì, T., Ottolini, D. & Carafoli, E. The plasma membrane calcium pump in health and disease. *FEBS J.* **280**, 5385–5397 (2013).
16. VanHouten, J. *et al.* PMCA2 regulates apoptosis during mammary gland involution and predicts outcome in breast cancer. *Proc. Natl. Acad. Sci. USA* **107**, 11405–11410 (2010).
17. Li, S. *et al.* PLXNA2 knockdown promotes M2 microglia polarization through mTOR/STAT3 signaling to improve functional recovery in rats after cerebral ischemia/reperfusion injury. *Exp. Neurol.* **346**, 113854 (2021).
18. Toledano, S. *et al.* Plexin-A2 enables the proliferation and the development of tumors from glioblastoma derived cells. *Cell Death Dis.* **14**, 1–13 (2023).
19. Andersen, J. V. *et al.* Glutamate metabolism and recycling at the excitatory synapse in health and neurodegeneration. *Neuropharmacology* **196**, 108719 (2021).
20. Lian, G. *et al.* Glutathione de novo synthesis but not recycling process coordinates with glutamine catabolism to control redox homeostasis and directs murine T cell differentiation. *eLife* **7**, e36158 (2018).
21. Sakiyama, T., Musch, M. W., Ropeleski, M. J., Tsubouchi, H. & Chang, E. B. Glutamine increases autophagy under basal and stressed conditions in intestinal epithelial cells. *Gastroenterology* **136**, 924-932.e2 (2009).
22. Palmieri, E. M. *et al.* Blockade of glutamine synthetase enhances inflammatory response in microglial cells. *Antioxid. Redox Signal.* **26**, 351–363 (2017).
23. Zhou, Y., Eid, T., Hassel, B. & Danbolt, N. C. Novel aspects of glutamine synthetase in ammonia homeostasis. *Neurochem. Int.* **140**, 104809 (2020).
24. Zitman, F. M. P. *et al.* Dentate gyrus local circuit is implicated in learning under stress—a role for neurofascin. *Mol. Neurobiol.* **53**, 842–850 (2016).
25. Zonta, B. *et al.* A critical role for Neurofascin in regulating action potential initiation through maintenance of the axon initial segment. *Neuron* **69**, 945–956 (2011).
26. Kriebel, M., Wuchter, J., Trinks, S. & Volkmer, H. Neurofascin: A switch between neuronal plasticity and stability. *Int. J. Biochem. Cell Biol.* **44**, 694–697 (2012).
27. Damal Villivalam, S. *et al.* A necessary role of DNMT3A in endurance exercise by suppressing ALDH1L1-mediated oxidative stress. *EMBO J.* **40**, e106491 (2021).
28. Jung, Y. *et al.* Epigenetic regulation of miR-29a/miR-30c/DNMT3A axis controls SOD2 and mitochondrial oxidative stress in human mesenchymal stem cells. *Redox Biol.* **37**, 101716 (2020).
29. Cobo, I., Tanaka, T., Glass, C. K. & Yeang, C. Clonal hematopoiesis driven by DNMT3A and TET2 mutations: role in monocyte and macrophage biology and atherosclerotic cardiovascular disease. *Curr. Opin. Hematol.* **29**, 1–7 (2022).
30. Leoni, C. *et al.* Dnmt3a restrains mast cell inflammatory responses. *Proc. Natl. Acad. Sci. USA* **114**, E1490–E1499 (2017).
31. Sue, N. *et al.* Targeted disruption of the basic krüppel-like factor gene (Klf3) reveals a role in adipogenesis. *Mol. Cell. Biol.* **28**, 3967–3978 (2008).
32. Knights, A. J. *et al.* Krüppel-like factor 3 (KLF3/BKLF) is required for widespread repression of the inflammatory modulator galectin-3 (Lgals3). *J. Biol. Chem.* **291**, 16048–16058 (2016).
33. Knights, A. J. *et al.* Krüppel-like factor 3 (KLF3) suppresses NF-κB–driven inflammation in mice. *J. Biol. Chem.* **295**, 6080–6091 (2020).

34. Kotlík, P. *et al.* Adaptive phylogeography: functional divergence between haemoglobins derived from different glacial refugia in the bank vole. *Proc. R. Soc. B* **281**, 20140021 (2014).
35. Strážnická, M., Marková, S., Searle, J. B. & Kotlík, P. Playing hide-and-seek in beta-globin genes: gene conversion transferring a beneficial mutation between differentially expressed gene duplicates. *Genes* **9**, 492 (2018).
36. Dvořáková, V., Horníková, M., Němcová, L., Marková, S. & Kotlík, P. Regulatory variation in functionally polymorphic globin genes of the bank vole: a possible role for adaptation. *Front. Ecol. Evol.* **7**, (2020).
37. Glaser, C., Heinrich, J. & Koletzko, B. Role of *FADS1* and *FADS2* polymorphisms in polyunsaturated fatty acid metabolism. *Metabolism* **59**, 993–999 (2010).
38. Pace, K. E., Hahn, H. P., Pang, M., Nguyen, J. T. & Baum, L. G. Cutting Edge: CD7 delivers a pro-apoptotic signal during galectin-1-induced T cell death. *J. Immunol.* **165**, 2331–2334 (2000).
39. Sempowski, G. D., Cross, S. J., Heinly, C. S., Scearce, R. M. & Haynes, B. F. CD7 and CD28 are required for murine CD4+CD25+ regulatory T cell homeostasis and prevention of thyroiditis. *J. Immunol.* **172**, 787–794 (2004).
40. Liang, S., Zheng, Y.-Y. & Pan, Y. Blood transcriptome analysis uncovered COVID-19–myocarditis crosstalk. *Microb. Pathog.* **189**, 106587 (2024).
41. Breuza, L. *et al.* Proteomics of endoplasmic reticulum-Golgi intermediate compartment (ERGIC) membranes from brefeldin A-treated HepG2 cells identifies ERGIC-32, a new cycling protein that interacts with human Erv46. *J. Biol. Chem.* **279**, 47242–47253 (2004).
42. Vainio, P. *et al.* High-throughput transcriptomic and RNAi analysis identifies AIM1, ERGIC1, TMED3 and TPX2 as potential drug targets in prostate cancer. *PLoS One* **7**, e39801 (2012).
43. Mohammad, I. *et al.* Flightless I is a focal adhesion-associated actin-capping protein that regulates cell migration. *FASEB J.* **26**, 3260–3272 (2012).
44. Kuwabara, Y. *et al.* A human FLII gene variant alters sarcomeric actin thin filament length and predisposes to cardiomyopathy. *Proc. Natl. Acad. Sci. USA* **120**, e2213696120 (2023).
45. Choi, S. S. *et al.* Flightless-1 inhibits ER stress-induced apoptosis in colorectal cancer cells by regulating $Ca^{2+}$ homeostasis. *Exp. Mol. Med.* **52**, 940–950 (2020).
46. Jin, J. *et al.* LRRFIP2 negatively regulates NLRP3 inflammasome activation in macrophages by promoting Flightless-I-mediated caspase-1 inhibition. *Nat. Commun.* **4**, 2075 (2013).
47. Li, J., Yin, H. L. & Yuan, J. Flightless-I regulates proinflammatory caspases by selectively modulating intracellular localization and caspase activity. *J. Cell Biol.* **181**, 321–333 (2008).
48. Chih, B. *et al.* A ciliopathy complex at the transition zone protects the cilia as a privileged membrane domain. *Nat. Cell Biol.* **14**, 61–72 (2012).
49. Dowdle, W. E. *et al.* Disruption of a ciliary B9 protein complex causes Meckel syndrome. *Am. J. Hum. Genet.* **89**, 94–110 (2011).
50. Villumsen, B. H. *et al.* A new cellular stress response that triggers centriolar satellite reorganization and ciliogenesis. *EMBO J.* **32**, 3029–3040 (2013).
51. Chen, T.-Y. *et al.* Genotoxic stress-activated DNA-PK-p53 cascade and autophagy cooperatively induce ciliogenesis to maintain the DNA damage response. *Cell Death Differ.* **28**, 1865–1879 (2021).
52. Pampliega, O. *et al.* Functional interaction between autophagy and ciliogenesis. *Nature* **502**, 194–200 (2013).
53. Alfaleh, M. A. *et al.* Extracellular matrix detached cancer cells resist oxidative stress by increasing histone demethylase KDM6 activity. *Saudi J. Biol. Sci.* **31**, 103871 (2024).
54. Nanduri, J., Wang, N., Wang, B. L. & Prabhakar, N. R. Lysine demethylase KDM6B regulates HIF-1α-mediated systemic and cellular responses to intermittent hypoxia. *Physiol. Genomics* **53**, 385–394 (2021).
55. Salminen, A., Kaarniranta, K., Hiltunen, M. & Kauppinen, A. Histone demethylase Jumonji D3 (JMJD3/KDM6B) at the nexus of epigenetic regulation of inflammation and the aging process. *J. Mol. Med.* **92**, 1035–1043 (2014).
56. Cheng, G. *et al.* Identification of PLXDC1 and PLXDC2 as the transmembrane receptors for the multifunctional factor PEDF. *eLife* **3**, e05401 (2014).
57. Capdevila-Busquets, E. *et al.* Breast cancer genes PSMC3IP and EPSTI1 play a role in apoptosis regulation. *PLoS One* **10**, e0115352 (2015).

58. Ko, L., Cardona, G. R., Henrion-Caude, A. & Chin, W. W. Identification and characterization of a tissue-specific coactivator, GT198, that interacts with the DNA-binding domains of nuclear receptors. *Mol. Cell. Biol.* **22**, 357–369 (2002).
59. Zangen, D. *et al.* XX ovarian dysgenesis is caused by a PSMC3IP/HOP2 mutation that abolishes coactivation of estrogen-driven transcription. *Am. J. Hum. Genet.* **89**, 572–579 (2011).
60. Pezza, R. J. *et al.* The dual role of HOP2 in mammalian meiotic homologous recombination. *Nucleic Acids Res.* **42**, 2346–2357 (2014).
61. Sultan, A. *et al.* Nuclear Tau, a key player in neuronal DNA protection. *J. Biol. Chem.* **286**, 4566–4575 (2011).
62. Violet, M. *et al.* A major role for Tau in neuronal DNA and RNA protection in vivo under physiological and hyperthermic conditions. *Front. Cell. Neurosci.* **8**, (2014).
63. Siano, G. *et al.* Tau modulates VGluT1 expression. *J. Mol. Biol.* **431**, 873–884 (2019).
64. Pinioti, S. *et al.* A metabolic gene survey pinpoints fucosylation as a key pathway underlying the suppressive function of regulatory T cells in cancer. *Cancer Immunol. Res.* **11**, 1611–1629 (2023).
65. Huo, W. *et al.* Triclosan activates c-Jun/miR-218-1-3p/SLC35C1 signaling to regulate cell viability, migration, invasion and inflammatory response of trophoblast cells in vitro. *BMC Pregnancy Childbirth* **22**, 470 (2022).
66. Zhang, Y., Zhang, N., Song, W., Yousuf, S. & Li, W. Ablation of the GDP-fucose transporter suppresses lung cancer cell proliferation and migration by reducing expression of PD-L1. *J. Cancer* **14**, 3295–3308 (2023).
67. Crowley, J. L., Smith, T. C., Fang, Z., Takizawa, N. & Luna, E. J. Supervillin reorganizes the actin cytoskeleton and increases invadopodial efficiency. *Mol. Biol. Cell* **20**, 948–962 (2009).
68. Chen, Y. *et al.* F-actin and myosin II binding domains in supervillin. *J. Biol. Chem.* **278**, 46094–46106 (2003).
69. Fang, Z. & Luna, E. J. Supervillin-mediated suppression of p53 protein enhances cell survival. *J. Biol. Chem.* **288**, 7918–7929 (2013).
70. Liu, H.-P. *et al.* Association of supervillin with KIR2DL1 regulates the inhibitory signaling of natural killer cells. *Cell. Signal.* **23**, 487–496 (2011).
71. Zhou, J. *et al.* Supervillin contributes to LPS-induced inflammatory response in THP-1 cell-derived macrophages. *Inflammation* **45**, 356–371 (2022).
72. Liu, H. *et al.* DNA damage signalling recruits RREB-1 to the p53 tumour suppressor promoter. *Biochem. J.* **422**, 543–551 (2009).
73. Chen, R.-L., Chou, Y.-C., Lan, Y.-J., Huang, T.-S. & Shen, C.-K. J. Developmental silencing of human ζ-globin gene expression Is mediated by the transcriptional repressor RREB1. *J. Biol. Chem.* **285**, 10189–10197 (2010).
74. Flajollet, S., Poras, I., Carosella, E. D. & Moreau, P. RREB-1 Is a transcriptional repressor of HLA-G1. *J. Immunol.* **183**, 6948–6959 (2009).
75. Hui, B. *et al.* RREB1-induced upregulation of the lncRNA AGAP2-AS1 regulates the proliferation and migration of pancreatic cancer partly through suppressing ANKRD1 and ANGPTL4. *Cell Death Dis.* **10**, 1–15 (2019).
76. Mehta, A. D. *et al.* Myosin-V is a processive actin-based motor. *Nature* **400**, 590–593 (1999).
77. Vogel, G. F. *et al.* Cargo-selective apical exocytosis in epithelial cells is conducted by Myo5B, Slp4a, Vamp7, and Syntaxin 3. *J. Cell Biol.* **211**, 587–604 (2015).
78. Roland, J. T. *et al.* Rab GTPase–Myo5B complexes control membrane recycling and epithelial polarization. *Proc. Natl. Acad. Sci. USA* **108**, 2789–2794 (2011).
79. Edlund, S. *et al.* Transforming growth factor-β1 (TGF-β)–induced apoptosis of prostate cancer cells involves Smad7-dependent activation of p38 by TGF-β-activated kinase 1 and mitogen-activated protein kinase Kinase 3. *Mol. Biol. Cell* **14**, 529–544 (2003).
80. Schiffer, M. *et al.* Apoptosis in podocytes induced by TGF-β and Smad7. *J. Clin. Invest.* **108**, 807–816 (2001).
81. Garo, L. P. *et al.* Smad7 controls immunoregulatory PDL2/1-PD1 signaling in intestinal inflammation and autoimmunity. *Cell Rep.* **28**, 3353-3366.e5 (2019).
82. Wang, W. *et al.* Signaling mechanism of TGF-β1 in prevention of renal inflammation: Role of Smad7. *J. Am. Soc. Nephrol.* **16**, 1371 (2005).
83. Kim, J.-H., Lane, W. S. & Reinberg, D. Human Elongator facilitates RNA polymerase II transcription through chromatin. *Proc. Natl. Acad. Sci. USA* **99**, 1241–1246 (2002).
84. Close, P. *et al.* Transcription impairment and cell migration defects in elongator-depleted cells: implication for familial dysautonomia. *Mol. Cell* **22**, 521–531 (2006).
85. Esberg, A., Huang, B., Johansson, M. J. O. & Byström, A. S. Elevated levels of two tRNA species bypass the requirement for elongator complex in transcription and exocytosis. *Mol. Cell* **24**, 139–148 (2006).

86. Karlsborn, T. *et al.* Elongator, a conserved complex required for wobble uridine modifications in Eukaryotes. *RNA Biol.* **11**, 1519–1528 (2014).
87. Dashzeveg, N., Taira, N., Lu, Z.-G., Kimura, J. & Yoshida, K. Palmdelphin, a novel target of p53 with Ser46 phosphorylation, controls cell death in response to DNA damage. *Cell Death Dis.* **5**, e1221–e1221 (2014).
88. Nie, Y. *et al.* Palmdelphin promotes myoblast differentiation and muscle regeneration. *Sci. Rep.* **7**, 41608 (2017).
89. Wang, S. *et al.* PALMD regulates aortic valve calcification via altered glycolysis and NF-κB–mediated inflammation. *J. Biol. Chem.* **298**, (2022).
90. Scheu, S. *et al.* Tspan2 is involved in anti-infectious immune responses and CNS autoimmunity. *J. Immunol.* **204**, 67.15 (2020).
91. de Monasterio-Schrader, P. *et al.* Uncoupling of neuroinflammation from axonal degeneration in mice lacking the myelin protein tetraspanin-2. *Glia* **61**, 1832–1847 (2013).
92. Miriyala, S. *et al.* Novel role of 4-hydroxy-2-nonenal in AIFm2-mediated mitochondrial stress signaling. *Free Radic. Biol. Med.* **91**, 68–80 (2016).
93. Lu, J. *et al.* Activation of *AIFM2* enhances apoptosis of human lung cancer cells undergoing toxicological stress. *Toxicol. Lett.* **258**, 227–236 (2016).
94. Nguyen, H. P. *et al.* Aifm2, a NADH oxidase, supports robust glycolysis and is required for cold- and diet-induced thermogenesis. *Mol. Cell* **77**, 600–617 (2020).
95. Leussis, M. P. *et al.* The *ANK3* bipolar disorder gene regulates psychiatric-related behaviors that are modulated by lithium and stress. *Biol. Psychiatry* **73**, 683–690 (2013).
96. Rangaraju, S. *et al.* Mood, stress and longevity: convergence on ANK3. *Mol. Psychiatry* **21**, 1037–1049 (2016).
97. Durak, O. *et al.* Ankyrin-G regulates neurogenesis and Wnt signaling by altering the subcellular localization of β-catenin. *Mol. Psychiatry* **20**, 388–397 (2015).
98. Zhou, D. *et al.* AnkyrinG is required for clustering of voltage-gated Na channels at axon initial segments and for normal action potential firing. *J. Cell Biol.* **143**, 1295–1304 (1998).
99. Kyriakakis, E. *et al.* T-cadherin attenuates the PERK branch of the unfolded protein response and protects vascular endothelial cells from endoplasmic reticulum stress-induced apoptosis. *Cell. Signal.* **22**, 1308–1316 (2010).
100. Joshi, M. B. *et al.* T-cadherin protects endothelial cells from oxidative stress-induced apoptosis. *FASEB J.* **19**, 1737–1739 (2005).
101. Denzel, M. S. *et al.* T-cadherin is critical for adiponectin-mediated cardioprotection in mice. *J. Clin. Invest.* **120**, 4342–4352 (2010).
102. Parker-Duffen, J. L. *et al.* T-cadherin Is essential for adiponectin-mediated revascularization. *J. Biol. Chem.* **288**, 24886–24897 (2013).
103. Popov, V. S. *et al.* T-cadherin deficiency Is associated with increased blood pressure after physical activity. *Int. J. Mol. Sci.* **24**, 14204 (2023).
104. Xin, W. *et al.* BCAT1 binds the RNA-binding protein ZNF423 to activate autophagy via the IRE1-XBP-1-RIDD axis in hypoxic PASMCs. *Cell Death Dis.* **11**, 1–16 (2020).
105. Casoni, F. *et al.* Zfp423/ZNF423 regulates cell cycle progression, the mode of cell division and the DNA-damage response in Purkinje neuron progenitors. *Development* **144**, 3686–3697 (2017).
106. Chaki, M. *et al.* Exome capture reveals ZNF423 and CEP164 mutations, linking renal ciliopathies to DNA damage response signaling. *Cell* **150**, 533–548 (2012).
107. Gupta, R. K. *et al.* Transcriptional control of preadipocyte determination by Zfp423. *Nature* **464**, 619–623 (2010).
108. Shao, M. *et al.* Zfp423 maintains white adipocyte identity through suppression of the beige cell thermogenic gene program. *Cell Metab.* **23**, 1167–1184 (2016).
109. Watanabe, T., Wang, S. & Kaibuchi, K. IQGAPs as key regulators of actin-cytoskeleton dynamics. *Cell Struct. Funct.* **40**, 69–77 (2015).
110. Clainche, C. L. *et al.* IQGAP1 stimulates actin assembly through the N-Wasp-Arp2/3 pathway. *J. Biol. Chem.* **282**, 426–435 (2007).
111. Fukata, M. *et al.* Regulation of cross-linking of actin filament by IQGAP1, a target for Cdc42. *J. Biol. Chem.* **272**, 29579–29583 (1997).
112. Birladeanu, A.-M. *et al.* The scaffold protein IQGAP1 links heat-induced stress signals to alternative splicing regulation in gastric cancer cells. *Oncogene* **40**, 5518–5532 (2021).
113. Yang, J. *et al.* MicroRNA-124 inhibits hepatic stellate cells inflammatory cytokines secretion by targeting IQGAP1 through NF-κB pathway. *Int. Immunopharmacol.* **95**, 107520 (2021).
114. Liu, H. *et al.* Beneficial role of erythrocyte adenosine A2B receptor-mediated AMPK activation in high altitude hypoxia. *Circulation* **134**, 405–421 (2016).
115. Oslund, R. C. *et al.* Bisphosphoglycerate mutase controls serine pathway flux via 3-phosphoglycerate. *Nat. Chem. Biol.* **13**, 1081–1087 (2017).

116. He, L., Ding, Y., Zhou, X., Li, T. & Yin, Y. Serine signaling governs metabolic homeostasis and health. *Trends Endocrinol. Metab.* **34**, 361–372 (2023).
117. Watanabe, M. *et al.* DOCK2 and DOCK5 act additively in neutrophils to regulate chemotaxis, superoxide production, and extracellular trap formation. *J. Immunol.* **193**, 5660–5667 (2014).
118. Lai, Y. *et al.* DOCK5 regulates energy balance and hepatic insulin sensitivity by targeting mTORC1 signaling. *EMBO Rep.* **21**, e49473 (2020).
119. Qu, H. *et al.* Dock5 deficiency promotes proteinuric kidney Ddseases via modulating podocyte lipid metabolism. *Adv. Sci.* **11**, 2306365 (2024).
