# Supplementary material for: Genetic admixture drives climate adaptation in the bank vole
Source: Commun Biol. 2024 Jul 15;7:863. doi: 10.1038/s42003-024-06549-z (PMC11251159; doi:10.1038/s42003-024-06549-z)
Supplement: Supplementary file 2 — Description of Additional Supplementary Files [file 42003_2024_6549_MOESM2_ESM.pdf]

Description of Additional Supplementary Files

File name: Supplementary Data 1
Description: Source data behind the graphs in Fig. 2.
