# Supplementary material for: Genetic admixture drives climate adaptation in the bank vole
Source: Commun Biol. 2024 Jul 15;7:863. doi: 10.1038/s42003-024-06549-z (PMC11251159; doi:10.1038/s42003-024-06549-z)
Supplement: Supplementary file 4 — Reporting summary [file 42003_2024_6549_MOESM4_ESM.pdf]

nature portfolio

Corresponding author(s): Petr Kotlík
Last updated by author(s): Jun 13, 2024

Reporting Summary

Nature Portfolio wishes to improve the reproducibility of the work that we publish. This form provides structure for consistency and transparency in reporting. For further information on Nature Portfolio policies, see our Editorial Policies and the Editorial Policy Checklist.

Statistics

For all statistical analyses, confirm that the following items are present in the figure legend, table legend, main text, or Methods section.

n/a	Confirmed	
☒	☐	The exact sample size (*n*) for each experimental group/condition, given as a discrete number and unit of measurement
☐	☒	A statement on whether measurements were taken from distinct samples or whether the same sample was measured repeatedly
☐	☒	The statistical test(s) used AND whether they are one- or two-sided<br>*Only common tests should be described solely by name; describe more complex techniques in the Methods section.*
☒	☐	A description of all covariates tested
☒	☐	A description of any assumptions or corrections, such as tests of normality and adjustment for multiple comparisons
☒	☐	A full description of the statistical parameters including central tendency (e.g. means) or other basic estimates (e.g. regression coefficient) AND variation (e.g. standard deviation) or associated estimates of uncertainty (e.g. confidence intervals)
☒	☐	For null hypothesis testing, the test statistic (e.g. *F*, *t*, *r*) with confidence intervals, effect sizes, degrees of freedom and *P* value noted<br>*Give P values as exact values whenever suitable.*
☐	☒	For Bayesian analysis, information on the choice of priors and Markov chain Monte Carlo settings
☒	☐	For hierarchical and complex designs, identification of the appropriate level for tests and full reporting of outcomes
☒	☐	Estimates of effect sizes (e.g. Cohen's *d*, Pearson's *r*), indicating how they were calculated

Our web collection on statistics for biologists contains articles on many of the points above.

Software and code

Policy information about availability of computer code

Data collection	The present study did not use algorithms or software that are central to the research but have not yet been described in the published literature.
Data analysis	The present study did not use algorithms or software that are central to the research but have not yet been described in the published literature.

For manuscripts utilizing custom algorithms or software that are central to the research but not yet described in published literature, software must be made available to editors and reviewers. We strongly encourage code deposition in a community repository (e.g. GitHub). See the Nature Portfolio guidelines for submitting code & software for further information.

Data

Policy information about availability of data

All manuscripts must include a data availability statement. This statement should provide the following information, where applicable:

- Accession codes, unique identifiers, or web links for publicly available datasets
- A description of any restrictions on data availability
- For clinical datasets or third party data, please ensure that the statement adheres to our policy

The SNP dataset of Marková et al. (ref. 49), from which the dataset used in this study was derived, as described in the corresponding Methods section, is accessible through the Dryad Digital Repository (https://doi.org/10.5061/dryad.kwh70rz96) (ref. 40).

Research involving human participants, their data, or biological material

Policy information about studies with human participants or human data. See also policy information about sex, gender (identity/presentation), and sexual orientation and race, ethnicity and racism.

Reporting on sex and gender	This study does not include human research participants.
Reporting on race, ethnicity, or other socially relevant groupings	This study does not include human research participants.
Population characteristics	This study does not include human research participants.
Recruitment	This study does not include human research participants.
Ethics oversight	This study does not include human research participants.

Note that full information on the approval of the study protocol must also be provided in the manuscript.

Field-specific reporting

Please select the one below that is the best fit for your research. If you are not sure, read the appropriate sections before making your selection.

☐ Life sciences ☐ Behavioural & social sciences ☒ Ecological, evolutionary & environmental sciences

For a reference copy of the document with all sections, see nature.com/documents/nr-reporting-summary-flat.pdf

Ecological, evolutionary & environmental sciences study design

All studies must disclose on these points even when the disclosure is negative.

Study description	Whole-genome sequencing data and genomic cline analyses were used to identify loci showing excess ancestry in a postglacial contact zone of the bank vole in Britain. These analyses were paired with genetic-environmental association tests to identify loci involved in climate adaptation to assess the importance of introgression in shaping local adaptation patterns.
Research sample	To assess the role of introgression in shaping the distribution of adaptive variation, the present manuscript uses a subset of SNP genotype data for bank voles (Clethrionomys glareolus) derived from a previous study (Marková et al. 2023 Nature Communications 14, 7840 https://doi.org/10.1038/s41467-023-43383-z). In that earlier study, we used the full dataset to determine the capability of local populations to adapt to current and future climatic conditions. In the current manuscript, we use genomic cline analysis to assess the extent to which local adaptation reflects adaptive introgression.
Sampling strategy	Data are from a previous study (see above). In that study, the sample size was set according to the available samples, aiming for a number of 10 individuals per population to ensure a balance between the number of individuals and localities.
Data collection	No new data were collected in this study.
Timing and spatial scale	Sequence data are from a previous study (see above), where they were obtained from available bank vole samples collected mainly between 2009 and 2011 in Britain.
Data exclusions	SNPs were filtered to remove loci with low coverage and quality and a high proportion of missing genotypes.
Reproducibility	Two independent bgc runs were performed to ensure convergence. Inferences about the role of introgression in adaptation are based on the concordant identification of adaptive loci by two different approaches.
Randomization	Because we were concerned with adaptation of local populations, random assignment of samples to groups was not relevant in our study.
Blinding	Blinding practice was not relevant to our study because no trials were performed.

Did the study involve field work? ☐ Yes ☒ No

Reporting for specific materials, systems and methods

We require information from authors about some types of materials, experimental systems and methods used in many studies. Here, indicate whether each material, system or method listed is relevant to your study. If you are not sure if a list item applies to your research, read the appropriate section before selecting a response.

Materials & experimental systems

n/a	Involved in the study
☒	☐ Antibodies
☒	☐ Eukaryotic cell lines
☒	☐ Palaeontology and archaeology
☒	☐ Animals and other organisms
☒	☐ Clinical data
☒	☐ Dual use research of concern
☒	☐ Plants

Methods

n/a	Involved in the study
☒	☐ ChIP-seq
☒	☐ Flow cytometry
☒	☐ MRI-based neuroimaging
